# Advancements in Inflammatory Bowel Disease: A Narrative Review of Diagnostics, Management, Epidemiology, Prevalence, Patient Outcomes, Quality of Life, and Clinical Presentation

**DOI:** 10.7759/cureus.41120

**Published:** 2023-06-28

**Authors:** Muhammad Ali Muzammil, FNU Fariha, Tirath Patel, Rohab Sohail, Munesh Kumar, Ejaz Khan, Bushra Khanam, Satesh Kumar, Mahima Khatri, Giustino Varrassi, Prasanthi Vanga

**Affiliations:** 1 Internal Medicine, Dow University of Health Sciences, Karachi, Karachi, PAK; 2 Medicine, Dow University of Health Sciences, Karachi, Karachi, PAK; 3 Medicine, American University of Antigua, St. John's, ATG; 4 Internal Medicine, Quaid-e-Azam Medical College, Bahawalpur, PAK; 5 Medicine, Liaquat University of Medical and Health Sciences, Jamshoro, PAK; 6 Dermatology, All India Institute of Medical Sciences, New Delhi, New Delhi, IND; 7 Internal Medicine, National Tuberculosis Center, Kathmandu, NPL; 8 Medicine and Surgery, Shaheed Mohtarma Benazir Bhutto Medical College, Karachi, PAK; 9 Medicine and Surgery, Dow University of Health Sciences, Karachi, Karachi, PAK; 10 Pain Medicine, Paolo Procacci Foundation, Rome, ITA; 11 Medicine, Konaseema Institute of Medical Sciences and Research Institute, Amalapuram, IND

**Keywords:** review article, crohn’s disease (cd), ulcerative colitis (uc), ibd, inflammatory bowel disease

## Abstract

Inflammatory bowel disease (IBD), encompassing ulcerative colitis (UC) and Crohn's disease (CD), is a chronic, immune-mediated disorder that impacts the gastrointestinal tract. Significant advancements in the diagnosis and treatment of IBD have been made during the past few decades, improving patient outcomes. This narrative review aims to provide an overview of recent developments in the diagnosis and treatment of IBD. Both from an evaluative and therapeutic standpoint, the management of IBD has undergone significant change. The standard of treatment for treating UC and CD patients has changed due to several medical developments. These developments include amino-salicylates, immunosuppressants, biological agents, and new therapeutics. The review also addresses the difficulties in applying these developments in clinical practice. Globally, the prevalence of IBD is rising, with Asia among the regions with the highest rates. These environments provide particular difficulties, such as poor disease knowledge, a lack of diagnostic services, and infectious IBD mimics. These issues must be resolved to diagnose and manage IBD in these populations accurately. New imaging modalities and other improvements in diagnostic methods have increased the precision and early identification of IBD.

To reduce problems and improve patient outcomes, healthcare professionals treating patients with IBD must work effectively as a team. An extensive summary of current developments in the diagnosis and treatment of IBD is given in this narrative review. It draws attention to the therapeutic possibilities, difficulties, and uncertainties of integrating these developments into clinical practice. By keeping up with these changes, healthcare practitioners can better care for patients with IBD and improve their quality of life.

## Introduction and background

Inflammatory bowel disease (IBD) is a chronic and debilitating inflammatory illness of the gastrointestinal tract that predominantly consists of two different entities: ulcerative colitis (UC) and Crohn's disease (CD). IBD pathogenesis is complicated and complex, involving genetic vulnerability, dysregulated immunological responses, and environmental factors [1,2]. The growing global prevalence of IBD and the significant burden it places on individuals and healthcare systems need ongoing advances in diagnosis and management [3]. Considerable progress has been made in understanding the pathophysiology of IBD during the last few decades. This increased understanding has resulted in the development of novel diagnostic techniques and therapeutic strategies to improve disease control and patient outcomes. Non-invasive imaging techniques such as magnetic resonance enterorrhaphy and capsule endoscopy, which allow for a more precise assessment of illness extent and severity, have been used in diagnostic improvements [1]. Furthermore, discovering new serological and genetic markers has improved diagnostic accuracy and prognostic assessment in IBD patients. In terms of management, the paradigm has moved from symptom-driven to treat-to-target, with the goal of mucosal healing and long-term remission [1]. The emergence of targeted biologic medicines that directly block important mediators of inflammation, such as tumor necrosis factor-alpha (TNF-α), interleukin (IL)-12, and IL-23, has aided this strategy [4]. Furthermore, advances in surgical techniques, such as minimally invasive procedures and laparoscopy, have resulted in better outcomes for patients requiring surgical intervention. Again, new research has shed light on IBD's extra-intestinal symptoms, such as its neurological, cardiovascular, and dermatological implications [5-7]. These discoveries have highlighted the significance of a multidisciplinary approach to IBD care, integrating gastroenterologists, rheumatologists, dermatologists, and other experts to address the disease's various clinical presentations.

In this narrative review, we hope to provide a complete summary of current advances in the diagnosis and therapy of IBD. We will review the most recent findings in the pathogenesis of IBD, emphasizing the roles of genetic and environmental variables. Furthermore, we will investigate the developing diagnostic modalities, such as imaging techniques and biomarkers, that aid in illness classification and monitoring. Moreover, we will look at the most current therapeutic methods, such as targeted biologics and surgical therapies, and how they affect disease outcomes. Furthermore, we will go through the extra-intestinal symptoms of IBD and how they affect patient care. Overall, this study aims to give clinicians and researchers a current awareness of developments in IBD diagnosis and management, allowing for better patient care and outcomes.

## Review

Epidemiology and prevalence of inflammatory bowel disease

**Figure 1:**
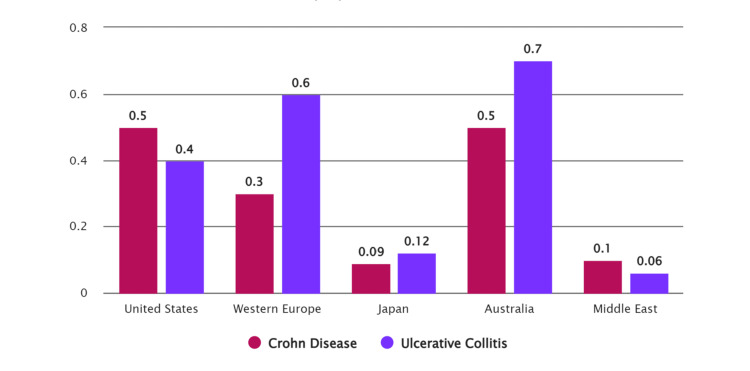
Prevalence and incidence of inflammatory bowel disease in different population/countries Source: References [8-10]

Understanding IBD epidemiology and prevalence is critical for accurate diagnosis and management. Based on pertinent studies, this section provides a complete summary of the epidemiology and prevalence of IBD, as shown in Figure 1.

A meta-analysis of population-based studies completed in the 21st century evaluated the global incidence and prevalence of IBD. The review found that the incidence and prevalence of IBD are growing globally, with the most significant rates seen in North America and Europe [8]. Another comprehensive analysis spanned several decades revealed a growing trend in the incidence and prevalence of IBD, including CD and UC, in several countries worldwide [9]. There are regional differences in IBD incidence and prevalence, particularly in Asia. A review concentrating on Asia found differences in IBD incidence and prevalence across the region's countries [10]. Furthermore, a study that examined data from 195 nations and territories between 1990 and 2017 found geographical disparities in the burden of IBD, emphasizing the need for targeted prevention and treatment efforts [11]. The epidemiology of IBD has evolved following Omran's Epidemiologic Transition Theory's four stages. IBD was once considered an illness that only affected Western nations but is now a global epidemic. In recent years, the prevalence of IBD has increased in newly industrialized countries [12]. Genetic predisposition, family history, and environmental variables are all risk factors for having IBD. The high concordance between dizygotic twins suggests a substantial genetic component in IBD risk. Furthermore, several ecological variables and their interactions with genetic vulnerability influence the disease burden of IBD [13]. Primary care-based cohort studies in the United Kingdom have shed light on the incidence and prevalence of IBD. These investigations have helped better understand IBD's temporal trends and prevalence in the UK population [14]. IBD is a worldwide illness with a rising incidence and prevalence. Regional variances exist, and the illness burden is changing. A combination of genetic and environmental factors causes IBD. IBD epidemiology is dynamic, necessitating continual monitoring and study to enhance diagnosis and management strategies.

Pathophysiology of inflammatory bowel disease

IBD is a chronic and debilitating gastrointestinal inflammatory illness characterized by recurring episodes of intestinal inflammation. IBD is caused by a complex and multifaceted combination of genetic, environmental, immunological, and microbiological factors. Extensive research has dramatically improved our understanding of IBD pathogenesis. Based on the current study, this response will provide a complete summary of the pathogenesis of IBD. CD and UC are the two primary subtypes of IBD. CD is distinguished by transmural inflammation that can affect any portion of the gastrointestinal system, whereas UC typically affects the colon and rectum, with mucosal layer inflammation [15]. In genetically vulnerable people, IBD etiology involves dysregulated immune responses to commensal gut microbiota.

IBD is influenced by genetic factors, with over 200 susceptibility loci discovered to date [16]. NOD2, ATG16L1, and IL23R gene variants contribute to the dysregulated immune response and decreased mucosal barrier function found in IBD [15]. Environmental variables are also crucial in the pathophysiology of IBD. Smoking, food, changes in the gut microbiota, and virus exposure can all influence illness development, progression, and severity [17]. The gut microbiota is of particular relevance since dysbiosis and changes in microbial diversity have been documented in IBD patients. These changes can result in an imbalance of preventive and pro-inflammatory microbial species, exacerbating intestinal inflammation [18]. A dysregulated interplay of the innate and adaptive immune systems characterizes the immune response in IBD.

In response to microbial antigens, innate immune cells such as macrophages, dendritic cells, and neutrophils release pro-inflammatory cytokines and chemokines. This results in the recruitment and activation of adaptive immune cells such as T and B cells. T helper 1 (Th1) and T helper 17 (TH17) cells play essential roles in the pathogenesis of IBD by secreting pro-inflammatory cytokines such as interferon-gamma (IFN-) and interleukin-17 (IL-17) [17]. A faulty regulatory immune response causes the persistent inflammation seen in IBD. Regulatory T cells (Tregs) and regulatory B cells (Bregs) assist in maintaining immunological tolerance and preventing excessive inflammation. In IBD, dysfunction or weakening of these regulatory cell populations contributes to prolonged inflammation and tissue damage [18]. Changes in the intestinal epithelial barrier integrity and mucus layer function have also been linked to IBD pathogenesis. Tight junction protein, mucin synthesis, and antimicrobial peptide secretion defects weaken barrier function, allowing luminal antigens to interact with immune cells and generate an inflammatory response [19].

A complex interplay of genetic vulnerability, environmental variables, dysregulated immune responses, changes in the gut flora, and many other factors complicates the pathophysiology of IBD.

Clinical presentation and diagnosis

Clinical Presentation

Clinical manifestations of IBD vary greatly, but frequent symptoms include gastrointestinal pain, diarrhea, rectal bleeding, weight loss, exhaustion, and fever [20]. These symptoms, however, are not exclusive to IBD and can occur in other illnesses as well [21].

Crohn's disease: From the mouth to the anus, CD can affect any region of the gastrointestinal tract. The terminal ileum and colon are the most implicated areas. CD is characterized by transmural inflammation, resulting in strictures, fistulas, and abscesses. CD can also cause extra-intestinal symptoms such as joint pain, skin rashes, and eye irritation [20].

Ulcerative colitis: UC is an inflammatory disease primarily affecting the colon and rectum, generally involving the rectum and expanding proximally in a continuous pattern. In UC, the inflammation is restricted to the mucosal layer. Bloody diarrhea, urgency, tenesmus, and stomach cramps are all symptoms of UC [20].

Diagnosis

IBD is diagnosed using a combination of clinical assessment, endoscopic procedures, radiologic imaging, and histological testing [22], as shown in Table 1. The following are important diagnostic steps.

**Table 1:** Diagnostic modalities in inflammatory bowel disease

S. No	Diagnostic Modality	Description
1.	Radiologic imaging Modalities (ultrasonography, magnetic resonance imaging, computed tomography)	CT scan can examine the colon and small intestine to look for abnormalities such as abscesses, strictures, fistulas, and thickening of the intestinal wall. MRI is used for the assessment of small bowel disease, including the detection of strictures and fistulas. Ultrasound is a non-invasive method for assessing intestinal wall thickness, finding abscesses, and diagnosing perianal illness.
2.	Serologic Markers	C-reactive protein (CRP), erythrocyte sedimentation rate (ESR), and antibodies including anti-Saccharomyces cerevisiae antibody (ASCA) and anti-neutrophil cytoplasmic antibody (ANCA) are serological markers that can help distinguish between CD and UC while also supporting indications of inflammation.
3.	Endoscopy	Endoscopy is a useful tool for evaluating and diagnosing IBD. It aids in the visualization of the gastrointestinal tract.
4.	Small Bowel Capsule Endoscopy	Capsule endoscopy allows for precise visualization and sample of the mucosa throughout the bowel.
5.	Double-Balloon Enteroscopy	The small intestinal mucosa can be visualized and sampled using double-balloon enteroscopy.
6.	Molecular Surrogates	Molecular surrogates are gaining importance in the diagnosis, differential diagnosis, and evaluation of IBD.
7.	Chromoendoscopy, Magnification Endoscopy, Confocal Endomicroscopy, and Spectroscopy	The diagnosis of colorectal neoplasia and visualization of the mucosa are aided by advanced endoscopic procedures.

Clinical assessment: For the initial assessment of IBD, a complete history, physical examination, and symptom evaluation are required. Family history, lifestyle factors, and medication history may also be helpful [23].

Laboratory tests: Blood tests, such as a complete blood count, inflammatory indicators (such as C-reactive protein and erythrocyte sedimentation rate), and specific antibody tests (e.g., anti-Saccharomyces cerevisiae antibodies), can help with the diagnosis and monitoring of IBD [24].

Endoscopy: The gold standard for diagnosing IBD is a colonoscopy with biopsy. It provides direct viewing of inflamed mucosa and targeted biopsies for histological evaluation. Esophagogastroduodenoscopy may be performed if upper gastrointestinal involvement is suspected [20].

Imaging studies: Radiologic imaging, such as computed tomography (CT) and magnetic resonance imaging (MRI), can provide further information about the degree of the disease, complications, and extra-intestinal symptoms. Imaging modalities are especially helpful in determining small bowel involvement in CD [25].

Histopathology: Histological analysis of biopsied tissue samples aids in the differentiation of CD and UC and eliminates the possibility of alternative causes of inflammation. Histopathology can reveal disease activity, mucosal healing, and the presence of dysplasia or cancer [24-30].

It is crucial to emphasize that IBD is a multifaceted condition that often requires the involvement of various medical specialists, including gastroenterologists, radiologists, and pathologists [31,32]. This is particularly evident in the diagnosis of ulcerative colitis and Crohn's disease, where established diagnostic criteria and classification systems such as the Montreal classification, as shown in Table 2.

**Table 2:** Diagnostic criteria for ulcerative colitis and Crohn's disease include classification systems such as Montreal classification

Diagnostic Criteria	Ulcerative Colitis	Crohn’s Disease
Clinical Presentation	Chronic symptoms such as constipation urgency, abdominal discomfort, and bloody diarrhea are among the symptoms that are persistent and recurrent.	Chronic Symptoms that are persistent and recurrent include fever, lethargy, weight loss, diarrhea, and stomach pain.
Endoscopic Findings	Continuously inflammation that is only present in the colon and rectum.	Transmural inflammation that may affect any area of the digestive system. commonly impacts the colon and terminal ileum, but it can also affect other organs.
Histopathology	Inflammatory infiltrates in the lamina propria as well as crypt deformation, cryptitis, and crypt abscesses may be visible in biopsy samples.	Transmural inflammation, granulomas, and intermittent gastrointestinal involvement can all be seen in biopsy samples.
Classification System	The Montreal classification system is mainly used to categorize the anatomical level of ulcerative colitis involvement. It encompasses subtypes such as: 1. Pancolitis 2. Left-sided colitis 3. Proctitis.	The Montreal classification system is largely used to categorize the severity of Crohn's disease based on: 1. The location 2. Behavior 3. Age of diagnosis 4. Progression of the disease, It contains classifications for the location (L1-L4), (B1-B3), age at diagnosis (A1-A3), and progression (P) of the disease.

Medical management of inflammatory bowel disease

IBD, which includes UC and CD, is an inflammatory condition that primarily affects the intestinal tract and is chronic and recurrent [33]. IBD is managed with a multifaceted strategy incorporating traditional and cutting-edge treatments. Based on the material that is currently accessible, this article gives a general review of the medical therapy of IBD. The cornerstone of the management of IBD is conventional medication. When UC and CD patients have low to moderate disease activity, amino-salicylates (5-ASA) are frequently utilized as the first-line therapy [34]. Because of their adverse side effects, corticosteroids are only used to temporarily induce remission in patients with moderate to severe illness [34]. Methotrexate and thiopurines (6-mercaptopurine and azathioprine) are immunomodulators utilized in maintenance therapy to lessen corticosteroid requirements [34]. IBD treatment has undergone a revolution thanks to biological medicines. Infliximab and adalimumab are examples of TNF-α inhibitors that effectively elicit and maintain remission in moderate to severe UC and CD [35]. Vedolizumab and ustekinumab, two more biologics that target various pathways, have also demonstrated effectiveness in treating IBD [35]. Tofacitinib and other Janus kinase (JAK) inhibitors have recently received approval to treat moderate to severe UC [35], as shown in Table 3. The management of IBD requires a comprehensive approach in addition to medication.

**Table 3:** Medication of inflammatory bowel disease

S. No	Medication Class	Example
1.	Amino salicylates (5-ASA)	Mesalamine, Sulfasalazine, Balsalazide
2.	Immunomodulators	Azathioprine, 6-Mercaptopurine, Methotrexate
3.	Corticosteroids	Prednisone, Prednisolone, Budesonide
4.	Biologic Agents - TNF-α	Infliximab, Adalimumab, Certolizumab Pegol
5.	Biologic Agents - Integrin	Vedolizumab, Natalizumab
6.	Biologic Agents - IL-12/23	Ustekinumab
7.	Janus Kinase (JAK) Inhibitors	Tofacitinib
8.	Antibiotics	Ciprofloxacin, Metronidazole
9.	Topical Agents	Rectal Mesalamine, Corticosteroid Enemas
10.	Targeted Therapies	JAK Inhibitors (other than tofacitinib), S1P Modulators, SMAD7 Antisense Oligonucleotide

Particularly in CD children under 18, nutritional therapy produces remission, including exclusive enteral feeding [33]. For treating disease and quality of life, lifestyle changes, including quitting smoking, managing stress, and exercising frequently, are also crucial [33].

IBD management in particular populations necessitates unique considerations. Due to age-related comorbidities and polypharmacy, elderly IBD patients pose particular hurdles. Disease management and reducing drug-related hazards should coexist in individualized treatment programs [36]. To balance the hazards of IBD medication and cancer management, patients with IBD and concurrent cancer must thoroughly assess their treatment options [37-41]. To improve patient outcomes, gastroenterologists and oncologists must work closely [42-48]. IBD is treated medically using an all-encompassing strategy that combines traditional medication, biological medicines, nutritional therapy, and lifestyle changes. Patients with moderate to severe IBD now see much better treatment outcomes thanks to the development of biologics.

However, individualized treatment strategies considering patient features and comorbidities are crucial to maximize therapeutic results and reduce dangers. For IBD management tactics to be improved and new therapeutic alternatives to be explored, more research and clinical trials are required for Surgical.

Complications and disease progression

IBD, also known as Crohn's disease and ulcerative colitis, is an immune-mediated condition that affects the gastrointestinal system and is chronic and progressive. IBD has a very variable course from person to person, and it is linked to several problems that might affect how the disease develops and how patients fare. Based on the mentioned references, I will summarize some of the IBD-related problems and illness progression variables in this response.

IBD problems fall into two categories: intestinal complications and extra-intestinal issues. Examples of intestinal issues include strictures, fistulas, bowel blockage, perforations, and colorectal malignancy [49]. Chronic inflammation, immunological dysregulation, and the digestive tract's healing process can all contribute to these issues. The consequences of strictures include bowel obstruction, symptoms such as abdominal pain and changed bowel habits, and a narrowing of the intestinal lumen. Fistulas are improper connections between sections of the intestine or between the gut and other organs, which frequently causes excrement or gas to travel through the intestine unusually. Surgery may be necessary to treat the symptoms and return to normal bowel function after these issues.

The gastrointestinal tract is not the only organ or system extra-intestinal consequences of IBD might impact. Hepatobiliary symptoms, arthritis, skin conditions, ocular manifestations, and a higher risk of thromboembolic events are a few examples [50]. IBD patients typically experience hepatobiliary consequences, including primary sclerosing cholangitis (PSC), chronic hepatitis, and cholangiocarcinoma [50]. PSC is the most prevalent biliary disorder linked to IBD, characterized by bile duct fibrosis and inflammation. These extra-intestinal signs frequently call for interdisciplinary treatment from dermatologists, gastroenterologists, hepatologists, and rheumatologists.

Several variables impact IBD disease development. Significant predictors of illness course include disease severity, location, and the degree of inflammation inside the gastrointestinal system. A higher risk of complications and a more aggressive course of the disease are linked to severe inflammation, substantial colon or small intestine involvement, and early age of onset [51]. Environmental triggers, dysregulated immune response, changes in the gut microbiota, and genetic predisposition to disease are additional factors that can affect disease progression [52]. Optimizing treatment plans and illness management requires identifying patients at a high risk of problems [53]. To help in predicting the course of IBD, predictive models integrating clinical, genetic, and immune response markers have been created [54].

An all-encompassing strategy is necessary for managing IBD complications and disease progression. Treatment goals include reducing inflammation, achieving and maintaining remission, and avoiding complications. Immunosuppressants, biological treatments, and corticosteroids are often prescribed drugs [55]. Surgical operations may occasionally be required to address issues including strictures, fistulas, or intestinal obstruction [49].

Patient outcomes and quality of life

Numerous research has looked at patient outcomes and quality of life in IBD. These studies seek to comprehend how IBD affects patients' well-being, assess different facets of quality of life, and pinpoint variables linked to favorable or unfavorable outcomes. Key quality-of-life comparisons in IBD were reviewed by a systematic study published in two parts [56]. Part I of the review concentrated on comparisons between diseases, such as IBD and other medically unwell groups, and IBD and the healthy population. Part II compared conditions within a single disorder, such as ulcerative colitis and Crohn's disease, and changes in quality of life over time. The review emphasized the value of gathering data on patients' experiences from general and IBD-specific quality-of-life measures. Patient-Reported Outcome-Based Evaluation (PROBE), a new standard of the quality of life for IBD patients, was the focus of another study [57]. This measurement evaluated the psychosocial impact of IBD using freely accessible assessment instruments. PROBE gave insights into the experiences and quality of life of people with IBD by including patient-reported outcomes. The quality of life of IBD patients was revealed to be influenced by favorable psychological aspects [58]. The study looked at how the quality of life varied depending on diagnosis, gender, state of therapy, and attachment preferences. Relationships between sociodemographic, clinical, and constructive psychological factors and life quality were also investigated. The results suggested that these variables influence the quality of life that IBD patients experience.

IBD has seen a rise in using patient-reported outcome measures (PROMs) [59]. PROMs are helpful for healthcare professionals and commissioners looking to improve patient-centered care because they enable the documentation of outcomes that matter most to patients. The International Consortium for Health Outcomes Measurement (ICHOM) has developed a standardized collection of PROMs for IBD, which supports a uniform method for evaluating patient outcomes. The relationship between psychological variables and patients with IBD's quality of life has also been researched [60]. The prevalence of depressive, anxiety, and stress symptoms was discovered to harm the course of the disease and the general quality of life. To improve patients' general quality of life, this study emphasized the significance of treating psychological well-being in IBD care. Health status and sociodemographic characteristics have been linked to changes in IBD patients' health-related quality of life (HRQOL) [61]. The study examined HRQOL using the Inflammatory Bowel Disease Questionnaire (IBDQ) and emphasized the need to consider these variables when assessing patients' well-being. Many different facets of patients' experiences have been illuminated by research on patient outcomes and quality of life in inflammatory bowel disease. This research investigated IBD's effect on the quality of life, created novel measures to evaluate patient-reported results, found connections between favorable psychological aspects and quality of life, and evaluated the impact of sociodemographic and health status variables. These findings advance knowledge of IBD and highlight the significance of a holistic approach to patient care that considers medical and psychosocial factors.

Future directions and emerging therapies

IBD, which includes UC and CD, is a group of chronic illnesses marked by intermittent intestinal inflammation. The necessity for ongoing research and development of new therapeutic strategies is highlighted by the fact that primary and secondary therapy failure rates in IBD are still high, despite the availability of current biologics and small molecules [62]. In the field of IBD, several potential directions and cutting-edge treatments are being investigated. One area of interest is creating disease-specific therapies based on a deeper comprehension of the etiopathogenesis of IBD. The mechanisms behind IBD are being clarified through research, and new therapeutic targets are being found [63]. IBD immunobiology research has improved the understanding of possible treatment targets and mechanisms of action. Future IBD therapy options may include immunomodulatory medicines, according to the study. To achieve long-term remission, these treatments modify the immunological response and reestablish immune tolerance. Examples include immune checkpoint inhibitors, cytokine-targeted therapies, and regulatory T cell-based therapeutics [64,65]. IBD management has been greatly enhanced using biologics, such as anti-TNF medications. More individualized therapy strategies, though, are required. Precision medicine advancements, such as the discovery of genetic and immunologic biomarkers, provide promise for personalizing treatments for specific individuals [66].

Additionally, the field of artificial intelligence (AI) is becoming more significant in treating IBD. Large datasets from electronic health records, genomes, proteomics, and imaging modalities can be analyzed by AI applications like machine learning algorithms to get important insights into illness diagnosis, prognosis, and therapy response [67]. Future directions in IBD management include refining existing treatment regimens and researching brand-new therapeutic approaches. This necessitates the development of new drug formulations, enhanced patient monitoring, and more effective medication administration methods [68]. Precision medicine, AI applications, immunomodulatory medicines, and disease-specific therapy are the future directions and developing medications to manage inflammatory bowel disease. Current treatment approaches are also optimized. These developments are meant to increase IBD care overall, boost treatment results, and lower treatment failure rates.

## Conclusions

IBD, which includes UC and CD, is an immune-mediated disorder that is chronic and progressive with high morbidity and risk of complications. There have been several significant developments in the diagnosis and treatment of IBD over the past few decades, all of which have improved patient outcomes and quality of life. The main developments in the diagnosis and treatment of IBD are outlined in this narrative review. The delay in diagnosis, which might have adverse effects, is one problem in managing IBD from a diagnostic standpoint. For improving patient outcomes, early diagnosis and prompt treatment initiation are essential. The diagnosis of IBD has been more reliable and effective thanks to advancements in diagnostic procedures like endoscopy, imaging modalities like computed tomography and magnetic resonance enterography, and biomarker tests like fecal calprotectin and C-reactive protein. Therapeutic methods have advanced significantly in the realm of IBD therapy. The development of biological medicines that target particular inflammatory pathways has complemented traditional therapy choices, such as amino-salicylates, immunosuppressants, and corticosteroids. Biologics make higher rates of clinical response and remission possible, including anti-TNF drugs, anti-integrin medications, and interleukin-12/23 inhibitors, which have revolutionized the treatment of moderate to severe IBD. In addition, more recent small molecule inhibitors, such as JAK inhibitors, have demonstrated potential in managing IBD. Precision therapy and personalized medicine are becoming increasingly important ideas in managing IBD. Individualized therapy options based on the patient's genetic profile are being investigated thanks to genetic breakthroughs and identifying specific genetic variations associated with IBD. This strategy tries to maximize the effectiveness of treatment, reduce side effects, and customize therapy to each patient's particular needs. The coronavirus disease 2019 (COVID-19) epidemic has also raised concerns over the treatment of IBD. At first, patients with IBD, especially those receiving immunosuppressive therapies, were thought to be at higher risk. However, new research indicates that most IBD treatments can be continued safely during the pandemic with suitable safeguards.

Improving inflammatory bowel disease diagnosis and treatment has greatly enhanced patient care and results. The efficiency and accuracy of IBD diagnosis have improved with cutting-edge diagnostic methods such as endoscopy, imaging modalities, and biomarker testing. With the development of biologics and small molecule inhibitors, therapeutic techniques have changed, providing tailored and efficient treatment choices. The idea of personalized medicine based on genetic profiles can potentially improve treatment. Evidence supports the continuation of most IBD medications with the proper precautions, notwithstanding the difficulties brought on by the COVID-19 pandemic. These developments add up to an overall improvement in the diagnosis, therapy, and management of IBD, giving people with this chronic ailment hope for better results.
